# Identification of Host-Plant Volatiles and Characterization of Two Novel General Odorant-Binding Proteins from the Legume Pod Borer, *Maruca vitrata* Fabricius (Lepidoptera: Crambidae)

**DOI:** 10.1371/journal.pone.0141208

**Published:** 2015-10-30

**Authors:** Jing Zhou, Na Zhang, Pan Wang, Shichang Zhang, Daiqin Li, Kaiyu Liu, Guoxiu Wang, Xiaoping Wang, Hui Ai

**Affiliations:** 1 Hubei Key Laboratory of Genetic Regulation and Integrative Biology, School of Life Sciences, Central China Normal University, Wuhan, 430079, China; 2 Key Laboratory of Insect Resource Utilization & Sustainable Pest Management of Hubei Province, College of Plant Science and Technology, Huazhong Agricultural University, Wuhan, 430070, China; 3 Department of Biological Sciences, National University of Singapore, 14 Science Drive 4, Singapore, 117543, Singapore; Institute of Zoology, CHINA

## Abstract

Chemoreception is a key feature in selection of host plant by phytophagous insects, and odorant-binding proteins (OBPs) are involved in chemical communication of both insects and vertebrates. The legume pod borer, *Maruca vitrata* Fabricius (Lepidoptera: Crambidae) is one of the key pest species of cowpea and widely distributed throughout tropical and subtropical regions, causing up to 80% of yield loss. In this study, we investigated the electrophysiological responses of female *M*. *vitrata* to floral volatiles from *V*. *unguiculata*. Seventeen electroantennogram-active compounds were identified from floral volatiles of *V*. *unguiculata* by coupled gas chromatography-electroantennography (GC-EAD) and gas chromatography-mass spectrometry (GC-MS). Then, we cloned two novel full-length GOBP genes (*MvitGOBP1* and *MvitGOBP2*) from the antennae of *M*. *vitrata* using reverse transcription PCR. Protein sequence analysis indicated that they shared high sequence similarity with other Pyralididae insect GOBPs and had the typical six-cysteine signature. Real-time PCR analysis indicated that *MvitGOBP1-2* mRNA was highly expressed in the antennae of female adult with several thousands-fold difference compare to other tissue. Next, the recombinant *MvitGOBP1-2* was expressed in *Escherichia coli* and purified using Ni ion affinity chromatography. Fluorescence binding assays demonstrated that MvitGOBP1-2 had different binding affinities with 17 volatile odorant molecules including butanoic acid butyl ester, limonene, 4-ethylpropiophenone, 1H-indol-4-ol, butanoic acid octyl ester and 2-methyl-3-phenylpropanal. In the field trapping experiment, these six floral volatiles could effectively attract female moths and showed significant difference compared with the blank lure. These results suggested that MvitGOBPs and the seventeen floral volatiles are likely to function in the olfactory behavior response of female moths, which may have played crucial roles in the selection of oviposition sites. The six compounds that we have identified from the volatiles of *V*. *unguiculata* may provide useful information for exploring efficiency monitoring and integrated pest management strategies of this legume pod borer in the field.

## Introduction

Olfactory chemoreception is a crucial capability for insects, guiding them to find food sources, mating partners and oviposition hosts. Insects have evolved a highly sensitive and sophisticated olfactory system, by which they are capable of detecting thousands of external volatile compounds including plant odorants and sex pheromones [1]. Volatile chemical signals from the host-plants provide important cues for various insects to detect and locate appropriate food resources for reproduction [2–7]. The legume pod borer, *Maruca vitrata* Fabricius (Lepidoptera: Crambidae) is one of the key pest species of cowpea and widely distributed throughout tropical and subtropical regions, causing up to 80% of yield loss [8, 9]. Our previous studies indicated that it was an oligophagous insect with a host range of legumes including *Vigna unguiculata*, *Lablab purpureus*, and female moths preferred to lay eggs on flower buds/flowers of the host plants [10]. There are growing evidences suggested that the odor mixtures derived from flower buds/flowers of the host-plants may play an important role in host discrimination and location for *M*. *vitrata* [11–12]. Due to the economic importance of the legume pod borers, various control strategy including insecticides against this pest have been taken in the field of southern China. However, improper and immoderate application of insecticides causes severe pesticide residues, which challenge the safety and quality of legume products in developing countries including China [13–15]. Therefore, it is necessary to develop alternative biological control strategies, such as pheromone-based pest control to replace chemical pesticides.

In Lepidoptera insects, pheromones and other semiochemicals are thought to be transported in the insect antennae by odorant-binding proteins (OBPs), which ferry the signals across the sensillum lymph to the olfactory receptors [16]. Odorant-binding proteins are a group of small, water-soluble molecules found in the sensillum lymph and transport the hydrophobic odorants to their receptors in the chemosensory neurons of insects [17, 18]. General odorant binding proteins (GOBPs) and the pheromone binding proteins (PBPs) are important parts of insect olfactory gene family, and sensitive olfaction is vital for phytophagous insects in host foraging. The GOBPs are mainly combined with general odorant molecules, such as plant volatiles, and the PBPs mainly bind pheromones, which are a blend of compounds emitted by females to attract male adults [19–21]. GOBPs are located in the olfactory sensilla and further classified as GOBP1 and GOBP2 [22, 23]. In general, GOBPs are expressed equally in the antennae of male and female moths, and show a high similarity in amino sequence, which suggests that they are involved in the reception of “general” odorants such as those from plants [24]. Volatile odorant signals from the host plant are usually thought to be mediated by GOBPs in sensillar lymph surrounding the olfactory receptors [21, 25]. Previous researches on various GOBP proteins from different insects and host-plant volatile components have indicated that they are necessary and essential in host discrimination and oviposition location in various insect species [18–26]. Among these GOBPs, AsteGOBP1 can bind a broad range of odorants from hosts and even involved in the blood-feeding behavior of the Asian malaria mosquito, *Anopheles stephensi* [18]. LstiGOBP2 derived from the Meadow Moth (*Loxostege sticticalis*) has been reported to have high binding affinities to most of the abundant host-plant volatiles that elicited strong electrophysiological responses [23]. In the field, *M*. *vitrata* like to lay eggs on flower buds/flowers of legume vegetables by detecting volatile odor molecules from host-plants, but molecular mechanisms of perception are not well clear. Identification of host-plant volatiles and physiological function analysis of olfactory proteins including GOBPs from *M*.*vitrata* will be significant to enhance efficiency monitoring and integrated control of this pest in the field.

Our previous studies have demonstrated that *M*. *vitrata* females have a preference for ovipositing on flowers buds/flowers of *V*. *unguiculata* and *L*. *purpureus* [10]. This leads us to hypothesize that *M*. *vitrata* females may use particular chemical cues from these flowers buds/flowers to find suitable oviposition sites. Thus, the identification of volatiles from flowers buds/flowers of host plant and the analysis of their fluorescence binding affinities for olfactory proteins will help to elucidate the molecular recognition mechanism used in selection of the location and oviposition of *M*. *vitrata*. In this study, volatile components from flower buds/flowers of *V*. *unguiculata* were identified and tested for electroantennogram response of *M*. *vitrata* females. Simultaneously, full-length *M*. *vitrata GOBP1-2* cDNA were cloned and expressed in *Escherichia coli* to explore their function and signal transduction mechanism of volatile odorant molecules. Moreover, the tissue expression patterns of *GOBP1-2* of *M*. *vitrata* were investigated by real-time quantitative PCR. Finally, we measured ligand-binding activities of two novel general odorant-binding proteins of *M*. *vitrata* with key floral volatiles using a fluorescence competitive binding assay.

## Materials and Methods

### Ethics Statement

The *M*. *vitrata* used in this study were collected from a natural population in the field of Cihui Farm (30°59´N, 114°06´E), Wuhan City, Hubei Province, China. All necessary permits were obtained for the described field studies. The agricultural bureau of Cihui Farm in Wuhan City issued the permission for our field studies at this site. The field studies did not involve endangered or protected species. Additionally, specimen of this moth was exhibited in the Museum of Huazhong Agricultural University. All experimental animal procedures including this pest were approved by the Institutional Review Board at Central China Normal University in China (CCNUIRB).

### Insect and host plant

The larvae of *M*. *vitrata* were collected from the host, cowpeas (*Vigna unguiculata*) and reared on an artificial diet [27] until they reached maturation in the laboratory with controlled environmental conditions (temperature: 26 ± 1°C; photoperiod: 14 L: 10 D; relative humidity: 60% ± 10%). All adult females used in this experiment had no prior exposure to host plant odor, and they were used only once [10]. Cowpeas were cultivated in the experimental field of Huazhong Agricultural University (30°28´N, 114°20´E, Wuhan City, Hubei Province, China). No insecticides were applied during the period of this study.

### Headspace collection

The plant volatiles were collected as described in earlier studies [7, 8]. Ten flower buds/flowers were cut from the cowpeas and immediately placed in a 2 L glass cylinder for extraction. The adsorbents (50 mg, Porapak Q, 80/100 mesh; Supelco, Bellefonte, PA, USA) were held between plugs of glass wool in a glass tube (0.5 cm × 10 cm). The collection of volatiles was extracted for 4 h and independently triplicated. Volatiles adsorbed on Porapak Q were eluted with 2 ml (4 × 0.5 ml) hexane. Extracted samples were concentrated to 50 μL by a slow stream of nitrogen and then stored in glass vials at -80°C until the later analyses.

### Coupled gas chromatography-electroantennography (GC-EAD)

Headspace collections were initially analyzed by an Agilent Technologies 7890A GC with a flame ionization detector (FID) coupled with an electroantennogram detector (Syntech, Hilversum, The Netherlands) followed the procedures as described by [28] with some modifications. The column was kept at 50°C for 1 min, increased to 200°C at a rate of 10°C/min and then increased to 240°C at a rate of 5°C/min, which was maintained for 5 min. The outlet of the GC column was split with a specific Electronic Pressure Control splitter (Agilent) to obtain the requested flow accuracy in a ratio of 1: 3 for the FID and the cut antenna. Volatile compounds eluting from the GC column were led to the mounted antenna through a heated (230°C) transfer line (TC-02, Syntech, Hilversum, the Netherlands). Each antenna was prepared by cutting both distal and basic segments and was immediately mounted on the holder using conductive gel (Spectra^®^ 360; Parker Laboratories, Fairfield, New Jersey, USA). Each sample was tested five times. Each tested antenna was derived from a different female and used only once. Synthetic chemicals used in the experiments were purchased from Sigma Chemical Co., J&K Scientific Ltd., Tokyo Chemical Industry Co., Sinopharm Chemical Reagent Co. and Aladdin Chemistry Co., respectively.

### Gas chromatography-mass spectrometry (GC-MS)

Headspace collections were also analyzed on an Agilent 7890A GC equipped with a DB-WAX capillary column (30 m × 0.25 mm × 0.25 μm; J & W Scientific, Folsom, CA, USA) and interfaced with an Agilent 5975C mass selective detector. Column and oven temperature programs were identical to those described above. Helium was used as a carrier gas (1.5 ml/min) and the purge valve was opened 1 min after injection. The inlet was maintained at 250°C in splitless mode. Compounds that repeatedly elicited antennal responses were identified by comparing the retention times of the respective synthetic standards and mass spectral fragmentation patterns in an MS database (NIST08.L).

### RNA extraction, cloning and sequencing

Total RNA was extracted from the antennae of female *M*. *vitrata* using an OMEGA E.Z.N.A TM Total RNA Kit (Omega, USA). First-strand cDNA was synthesized by a Prime Script first-strand cDNA synthesis kit (Invitrogen, USA) according to the manufacturer’s instructions. The open reading frame (ORF) of *MvitGOBP1-2* (GenBank accession numbers: KT803048 and KJ143717.1) was amplified by PCR with gene specific primers (MvitF1, MvitR1, MvitF2 and MvitR2, Table 1). PCR products were sequenced following insertion into T_1_ vector (TransGen Biotech., China).

**Table 1:** Primers used in the experiments.

Primer name	Sequence (5’-3’)
MvitF1	ATGGCGGGCTGGAGGCTG
MvitR1	CTATGCCTCGCTCTGCATG
GOYF1	GCTGACGGAGGATAAGATGGA
GOYR1	CGGTGAGCAGGTTGAAGTAGC
GOBP1F	CATGCCATGGCTATGGCGGGCTGGAGGCTG
GOBP1R	CCGCTCGAGCTATGCCTCGCTCTGCATG
MvitF2	ATGCTGTCCATGTGGTATTTCG
MvitR2	TTAGTATTTCTCCATGACGGCCTCG
GOYF2	TGTCCAACAAGTTCTCCCTCC
GOYR2	AGCACGCAGCCACCTTCAC
GOBP2F	CCGGAATTCATGATGGCCAAGGTGAAAGC
GOBP2R	CCGCCGATTAGCTTCAGTTGCACCAACAC
ActinF	AGCACGGTATCATCACCAACT
ActinR	GGTCTCAAACATGATCTGGGT

### Expression pattern of *MvitGOBP1-2*

Real-time PCR was used to investigate the transcript levels of *MvitGOBP1-2* in different tissues. Total RNA was prepared in triplicate using Trizol (Omega, USA) and the genomic DNA was digested with RNA-free DNase. Four primers (GOYF1, GOYR1, GOYF2 and GOYR2, Table 1) were used to determine the relative abundance of *MvitGOBP1-2* mRNA. Real-time relative quantitative reverse transcription polymerase chain reaction was performed on Bio-Rad CFX 96 real-time PCR system with SYBR Green I fluorescent dye. To check reproducibility, each real-time PCR reaction for each sample was carried out in three biological replicates and three technical biological replicates. Real-time PCR was conducted in 20 μL reactions that contained 10 μL of 2×TransStart Top Green qPCR SupperMix, 0.3 μL of each primer, 2 μL of sample cDNA and 7.4 μL ddH_2_O. The cycling conditions were: 95°C for 3 min; 40 cycles of 95°C for 10 s, 50°C for 30 s; Melt Curve 65°C to 95°C for 5 s.

### Recombinant expression of *MvitGOBP1-2*

The coding region of *MvitGOBP1-2* was amplified by polymerase chain reaction with specific primers (GOBP1F, GOBP1R, GOBP2F and GOBP2R, Table 1). The PCR product was cloned into pEASY-T1 vector (TransGen Biotech, China). The fragment was digested by two restriction enzymes *EcoR I* / *Xho I* and subsequently cloned into pET-32a (+) expression vector. The recombinant plasmid pET-32a(+) / *MvitGOBP1-2* was transformed into *E*. *coli* BL21 (DE3) competent cells. After a 3 h preincubation period, recombinant *MvitGOBP1-2* was induced with the addition of IPTG to a final concentration of 0.5 mM for 4 h. The bacteria were resuspended with PBS and broken using high pressure. Following centrifugation, MvitGOBP1-2 was purified from the supernatant using Ni ion affinity chromatography (Thermo, USA), and enterokinase was used to remove the His-tag. The size and purity of MvitGOBP1-2 were verified by SDS-PAGE analysis.

### Fluorescence binding assays

Fluorescence binding activity was determined as described by previous study [29]. Emission fluorescence spectra were recorded on a Hitachi F-4500 at 25°C in a right angle configuration, with a 1 cm light path quartz cuvette and 5 nm slits for both excitation and emission. The protein was dissolved in 20 mM Tris-HCl buffer, pH 7.4, and ligands were added as 1 mM methanol solutions. To measure the affinity of the fluorescent ligand 1-NPN to MvitGOBP1-2, a 2 μM protein solution in 20 mM Tris-HCl (pH 7.4) was titrated with aliquots of 1 μM ligand in methanol to final concentrations of 2–16 μM. The probe was excited at 337 nm and emission spectra were recorded between 380 and 450 nm. The affinities of the other ligands were measured in competitive binding assays, where a solution of the protein and 1-NPN, both at a concentration of 2 μM, was titrated with 1 mM methanol solutions of each competitor over concentration ranges of 2–24 μM, depending on the ligand. The dissociation constant for 1-NPN and the stoichiometry of binding were obtained by processing the data using Prism software. Dissociation constants of the competitors were calculated from the corresponding IC_50_ values (concentrations of ligands halving the initial fluorescence value of 1-NPN), using the equation: Ki = [IC_50_] / (1 + [1-NPN] / K_1-NPN_), where [1-NPN] is the free concentration of 1-NPN and K_1-NPN_ is the dissociation constant of the complex protein/1-NPN.

### Field Experiments

Field trap experiments were determined as described by Wang et al [30]. Custom-built Delta-traps with sticky inserts and rubber septa purchased from Pherobio Technology Co. Ltd. were used in the field experiments during the moth flight season in 2014. Traps were suspended from iron stakes and placed approximately 15 m apart. Key components of host-plant volatiles were prepared in hexane, and 100 μl solution (100 ng/μl) were added to rubber septa to be used as lures. Hexane was used as blank control and six trap replicates were used in the field experiment. Traps were checked everyday and the number of female moths per trap was determined for one week. Statistical analyses of trap data were used SPSS (SPSS Inc., Chicago, Illinous, U.S.A.), the significance of the differences between tested group and control group was evaluated by Student's t-test at *P <* 0.05 and *P <* 0.01.

## Results

### Identification and EAG responses of volatiles from *V*. *unguiculata*

**Fig 1:**
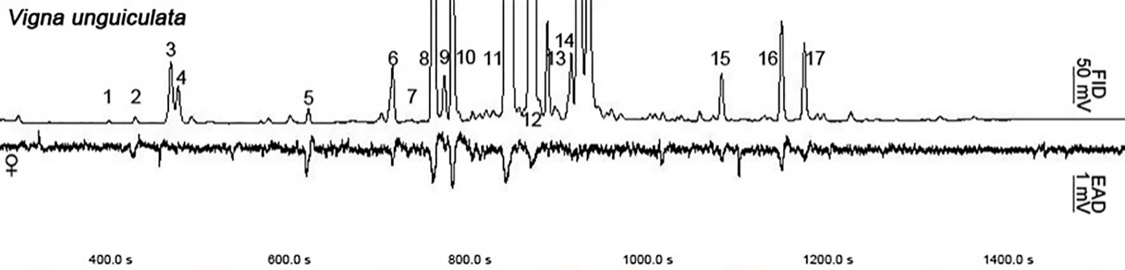
Gas chromatography-electroantennographic detection (GC-EAD) using the antennae of *Maruca vitrata* females in response to volatiles collected from cowpea (*Vigna unguiculata*) flower buds/flowers. The upper trace represents the flame ionization detector (FID) response and the lower trace represents the female-consistent antennal response (EAD). Chemical names corresponding to the peak numbers (1–17) are given in Table 1.

We have identified 17 major compounds from the volatile of *V*. *unguiculata* via GC-EAD analysis, which have elicited obvious EAG responses from *female M*. *vitrata* (Fig 1, Table 2). The antennae of *M*. *vitrata* females not only responded to the abundant compounds such as 4-ethylbenzaldehyde (8), 1-(4-ethylphenyl)-ethanone (10), 2-methyl-3-phenylpropanal (11) and 1-(2,4-Dimethylphenyl)-ethanone (12) but also to the less-abundant compounds, such as butanoic acid butyl ester (1), limonene (2), benzaldehyde (5), butanoic acid octyl ester (9) and 1H-indol-4-ol (15) (Fig 1, Table 2). Moreover, 2-Methyl-3-phenylpropanal was the most abundant compound and 1-(4-ethylphenyl)-ethanone in *V*. *unguiculata* elicited the highest EAD responses (Fig 1).

**Table 2:** The binding constants of different ligands. Binding of 1-NPN and different ligands to MvitGOBP1-2. Note: IC_50_, ligand concentration displacing 50% of the fluorescence intensity of the MvitGOBPs /N-phenyl-1-naphthylamine complex; *Ki*, dissociation constant. Odorant concentrations that exceeded 50 μM for half-maximal inhibition are represented as ‘-’ and were not used for calculating *Ki* values.

No.	Compounds	MvitGOBP1		MvitGOBP2	
		IC_50_(μM)	*K*_i_(μM)	IC_50_(μM)	*K*_i_(μM)
1	Butanoic acid butyl ester	16.84	12.52	44.81	29.67
2	Limonene	18.57	13.81	44.14	29.23
3	1,3-diethylbenzene	38.72	28.81	33.83	22.4
4	1,4-diethylbenzene	25.83	19.21	40.92	27.09
5	Benzaldehyde	22.35	16.63	-	-
6	Acetophenone	28.49	21.2	40.04	26.51
7	2-hydroxybenzaldehyde	20.82	15.49	41.88	22.87
8	4-ethylbenzaldehyde	19.08	14.19	49.96	33.08
9	Butanoic acid octyl ester	36.06	26.83	12.87	8.5
10	1-(4-ethylphenyl)-ethanone	22.92	17.05	44.96	29.77
11	2-methyl-3-phenylpropanal	23.89	17.77	6.98	3.81
12	1-(2,4-Dimethylphenyl)-ethanone	24.21	18.01	-	-
13	4-ethylpropiophenone	20.44	15.21	39.33	26.04
14	4-hydroxy-3-methylacetophenone	24.56	18.27	25.79	17.07
15	1H-indol-4-ol	14.62	10.87	-	-
16	1,4-diacetyl benzene	30.54	22.72	49.63	32.86
17	1-(1,1-Dimethylethyl)-3,5-dimethylbenzene	22.56	16.78	-	-

### Amino acid sequence analysis of MvitGOBP1-2 and alignment to homologs of other species

**Fig 2:**
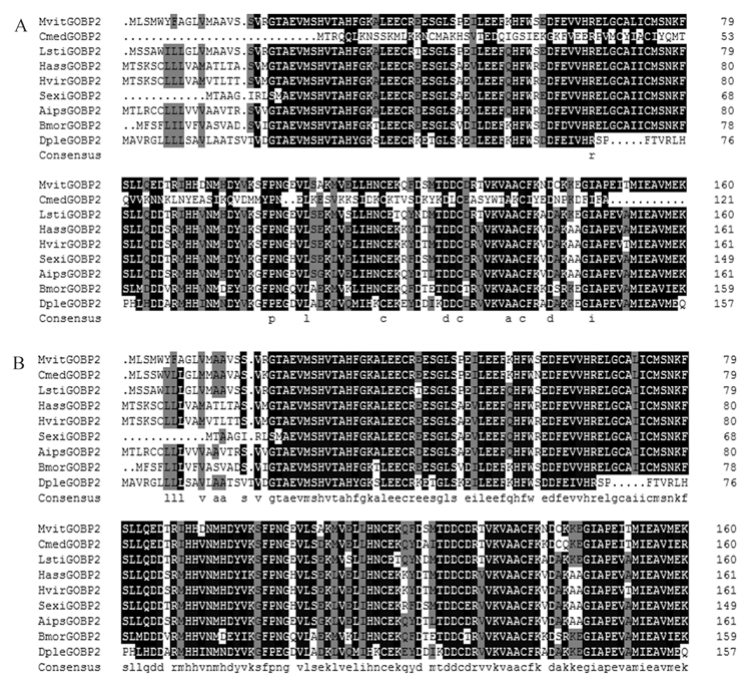
Alignment of GOBPs from Lepidopteran insects. MvitGOBP1 (A) and MvitGOBP2 (B) is aligned with the GOBPs of other Lepidopteran moths including CmedGOBPs (AFG72997.1), LstiGOBPs (ABY75632.1), HassGOBPs (AAQ54909.1), HvirGOBPs(CAA65606.1), SexiGOBPs (CAC12832.1), BmorGOBPs (CAA64445.1), DpleGOBPs (EHJ71306.1).

**Fig 3:**
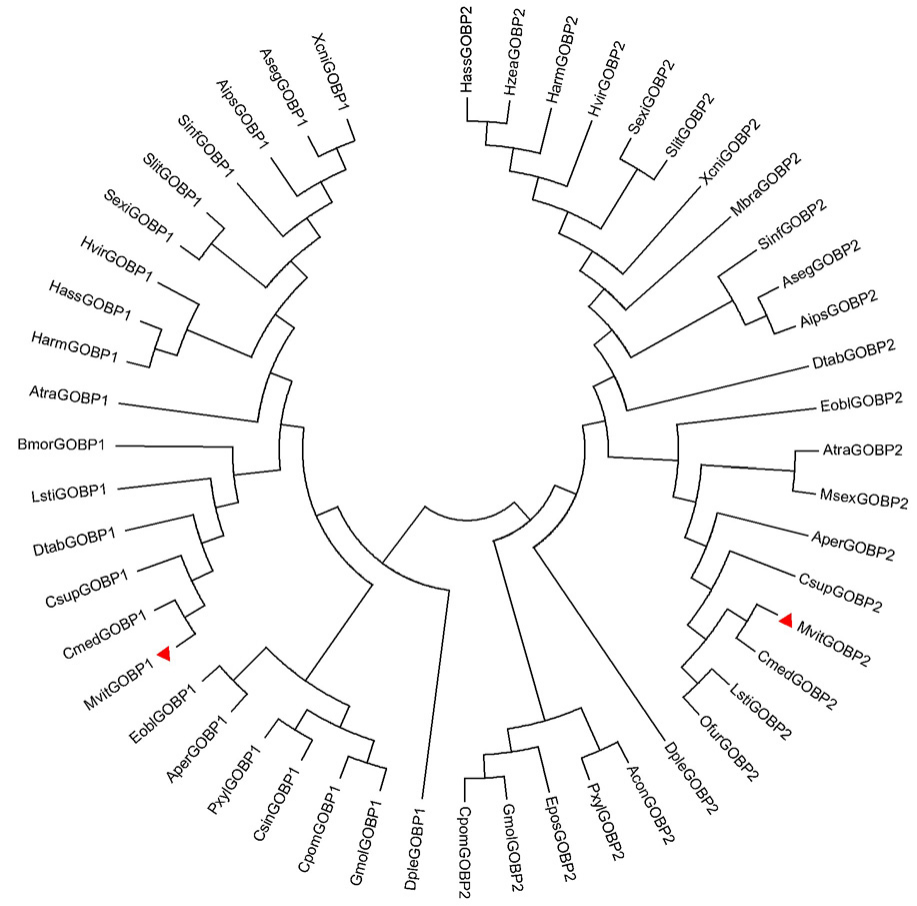
Phylogenetic tree of MvitGOBP1-2 amino acid sequence with those of GOBPs from other insect species. GenBank accession numbers: AipsGOBP1-2 (AFM367591, AFM367601), AsegGOBP1-2 (ABI241591, ABI241611), AtraGOBP1-2 (ACX478931, ACX478941), AperGOBP1-2 (CAA718661, CAA655751), AconGOBP2 (AFD341811), OfurGOBP2(ABG664192), CsupGOBP1-2 (ACJ071291, ACJ071201), CmedGOBP1-2 (AFG729961, AFG729971), CsinGOBP1 (AHY864931), BmorGOBP1 (CAA644441), CpomGOBP1-2 (AFP669571, AFP669581), DpleGOBP1-2 (EHJ713011, EHJ713061), DtabGOBP1-2 (AGJ712761, AGJ712771), EoblGOBP1-2 (ACN296801, ACN296811), EposGOBP2 (AAL058691), HzeaGOBP2 (AAG540781), GmolGOBP1-2 (AFH028411, AFH028421), HarmGOBP1-2 (AAL098211, CAC082111), HassGOBP1-2 (AAW650761, AAQ549091), HvirGOBP1-2 (CAA656051, CAA656061), LstiGOBP1-2 (ACB47481.1, ABY756321), MbraGOBP2 (AAC057032), MsexGOBP2 (AAG500151), PxylGOBP1-2 (ABY710341, ABY710352), SinfGOBP1-2(AGS367421, AHC723801), SexiGOBP1-2 (ACY784121, CAC128321), SlitGOBP1-2 (ABV321681, ABV321671), XcniGOBP1-2 (AGS414981, AGS414991).

Based on antenna transcriptome analyses of *M*. *vitrata*, full length cDNA encoding *MvitGOBPs* was cloned from *M*. *vitrata*. Both of MvitGOBP1 and MvitGOBP2 are deduced to be 161 amino acid protein encoded by 486 nucleotides (Fig 2A and 2B). The calculated molecular weight of MvitGOBP1 and MvitGOBP2 were 18.40 kDa and 18.36 kDa, respectively. The initial 17 and 20 amino acid residues were predicted to be a signal peptide of MvitGOBP1 and MvitGOBP2. An alignment of the deduced GOBP1-2 amino acid sequence from *M*. *vitrata* and other species of Lepidoptera is shown in Fig 2. Amino acid sequence analysis of MvitGOBP1-2 indicated that they shared high sequence identity (between 77% and 89%) with orthologs of other pyralididae insects and had the typical six-cysteine signature, which was very similar to other Lepidopteran GOBPs. Moreover, MvitGOBP1-2 exhibited the highest identity with CmedGOBP1-2 of *Cnaphalocrocis medinalis*, which was in accordance with their phylogenetic relationships (Fig 3). These results demonstrated that GOBPs were highly conserved among Lepidopteran species.

### Expression patterns of *MvitGOBPs*

**Fig 4:**
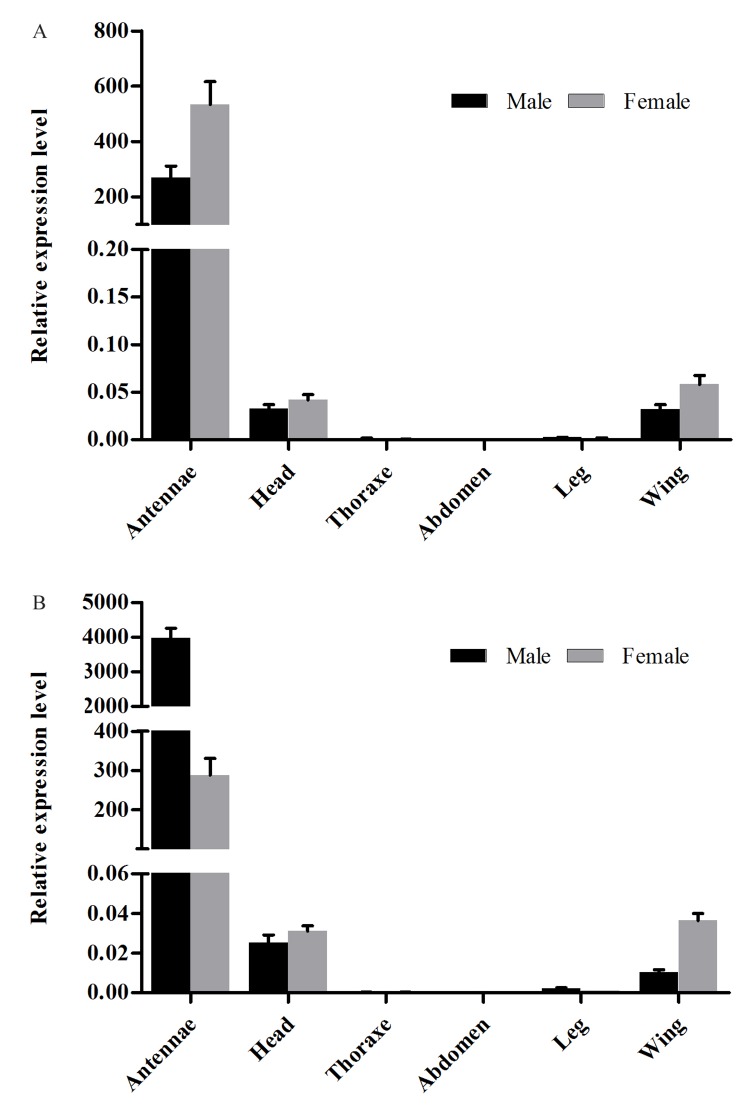
Expression levels of *MvitGOBP1-2* in different tissues measured by real-time qPCR.

Quantitative real-time PCR method was used to measure tissue-specific mRNA expression pattern of *MvitGOBP* genes (*MvitGOBP1* and *MvitGOBP2*). Transcript abundance for each GOBP was determined for multiple tissues (antenna, heads, thoraxes, abdomens, legs, wings) from adults of *M*. *vitrata*. The expression profiles analysis revealed that *MvitGOBP1-2* was predominantly expressed in antennae and the levels of transcripts were very low in other tissues (Fig 4A and 4B). Transcript level of *MvitGOBP1* in the male antennae is higher than that of female *M*. *vitrata*, but *MvitGOBP2* gene was sex-biased and specially expressed in male antenna with 13.78-fold difference compare to female moths.

### Expression and purification of MvitGOBP1-2

**Fig 5:**
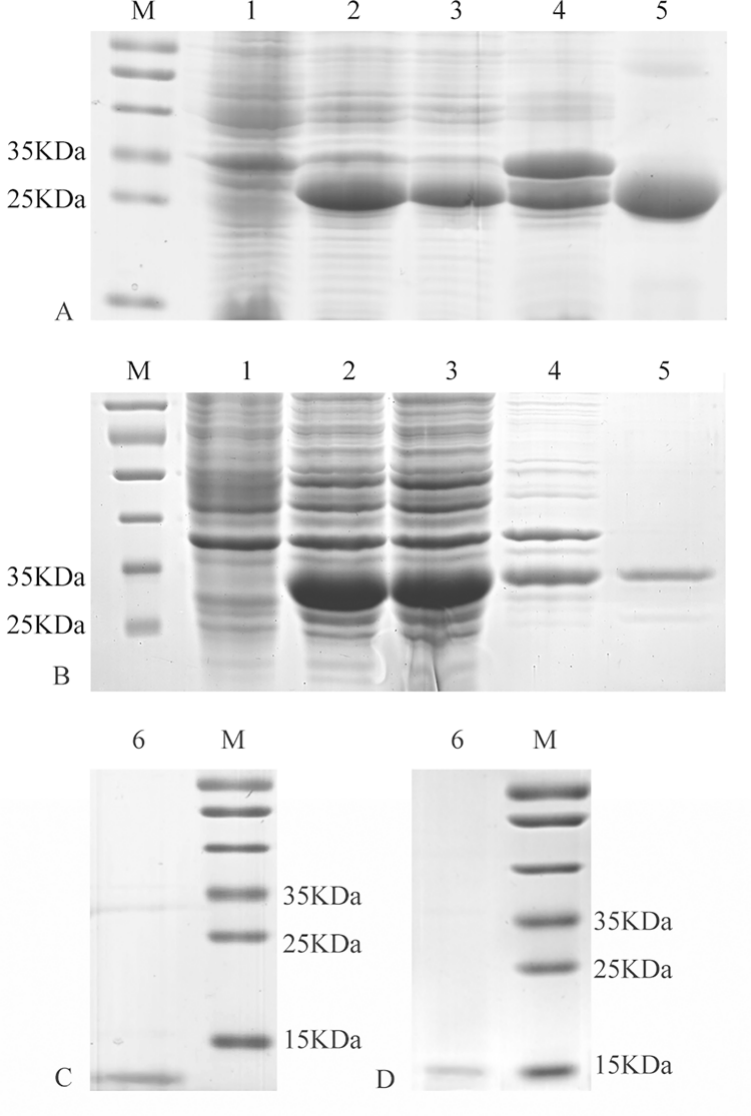
SDS-PAGE electrophoresis of recombinant MvitGOBP1 (A, C) and MvitGOBP2 (B, D). Lane 1—Non-induced *E*. *coli* MvitGOBP1-2; Lane 2- Induced *E*. *coli* MvitGOBP1-2, Lane 3—Supernatant after broken, Lane 4—Precipitation after broken, Lane 5—Purified MvitGOBP1-2 with His tag, Lane 6—Purified MvitGOBP1-2 without His tag, Lane M—Marker protein.

The recombinant plasmid pET-32a(+) / *MvitGOBPs* was transformed into competent *E*. *coli* BL21 (DE3) cells for expression of recombinant MvitGOBPs. The target protein was soluble, and expression was induced with 0.5 mM IPTG and purified with Ni-NTA resin after ultrasonication (Fig 5A and 5B). The target protein underwent two rounds of purification: the first round purified the recombinant protein from total protein, and then the His-tag of the recombinant MvitGOBP1-2 was removed by enterokinase (Fig 5C and 5D). Recombinant MvitGOBP1-2 was stored at—80°C until use in the binding experiment.

### Fluorescence binding affinities

**Fig 6:**
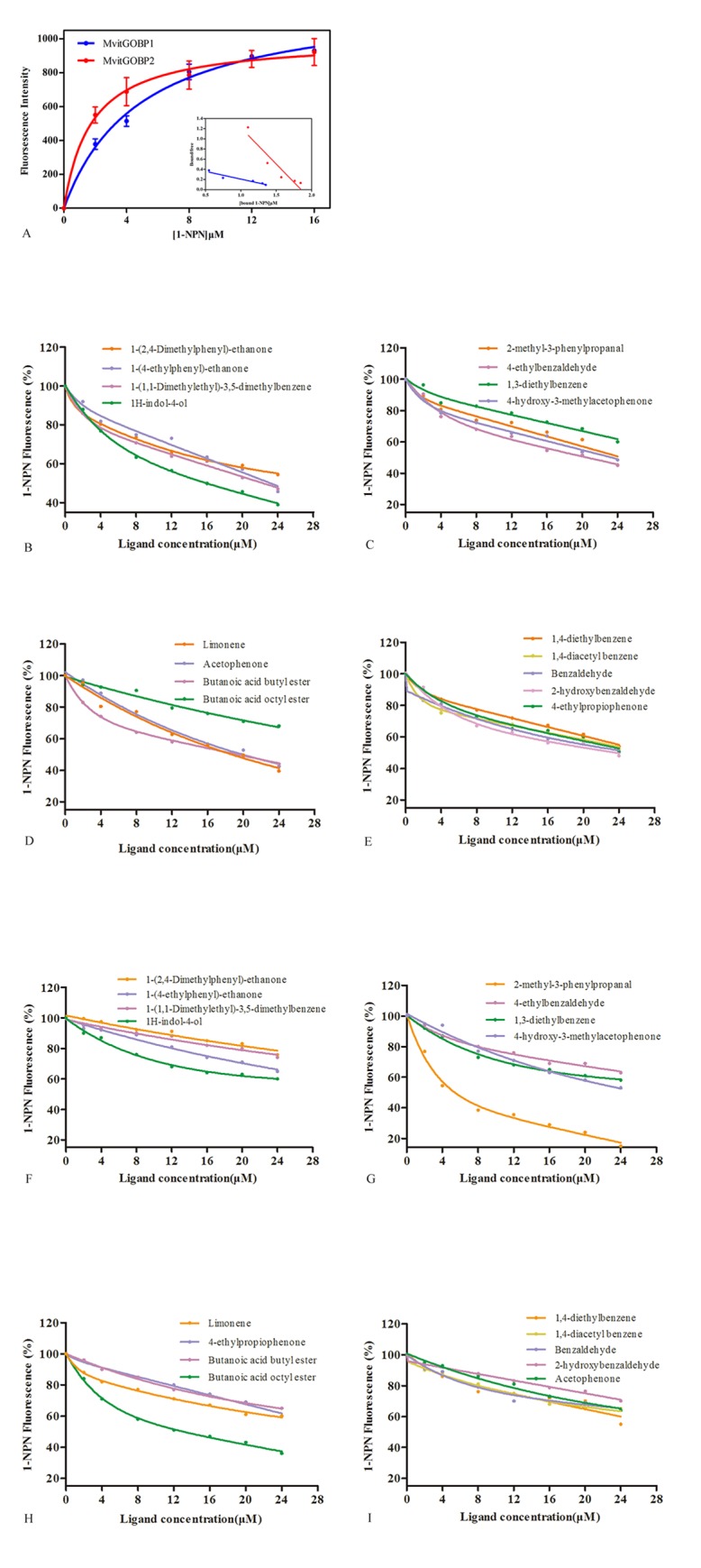
Ligand-binding experiments. (A) Binding curve and relative Scatchard plot. (B, C, D, E) Competitive binding curves of different ligands to the MvitGOBP1. (F, G, H, I) Competitive binding curves of different ligands to the MvitGOBP2.

Seventeen synthetic potential ligand chemicals exhibiting antenna electroantennogram response in floral volatiles were tested in competitive binding assays. As shown in Fig 6A, the binding curves and Scatchard plots indicated that the binding of the fluorescent ligand to both of MvitGOBPs increases with increasing concentrations of the 1-NPN. The IC_50_ values (the concentration of ligand halving the initial fluorescence values) and the calculated inhibition constants (*Ki*) where possible for each MvitGOBPs/ligand combination are shown in Table 2. Most of the tested volatiles succeeded in displacing 1-NPN from the MvitGOBPs/1-NPN complex at the concentrations up to 24 μM. The binding results demonstrated that MvitGOBP1 had high binding affinities with butanoic acid butyl ester, limonene, 4-ethylpropiophenone and 1H-indol-4-ol from the floral volatile components, which *Ki* values were 12.52, 13.81, 14.19 and 10.87 μM, respectively. Besides, when the concentration of butanoic acid octyl ester and 2-methyl-3-phenylpropanal reached 8.5, 3.81 μM, respectively, the fluorescence intensity of MvitGOBP2/1-NPN complex rapidly decreased to approximately 50%. And other floral volatile compounds also showed different binding abilities to MvitGOBP1 and MvitGOBP2, respectively (Fig 6B–6I and Table 2). Based on this, MvitGOBP1-2 showed robust binding affinities to partial floral volatiles, suggesting that MvitGOBPs play a key role in odorant signal transduction of *M*. *vitrata* recognize oviposition hosts.

### Field experiments

**Fig 7:**
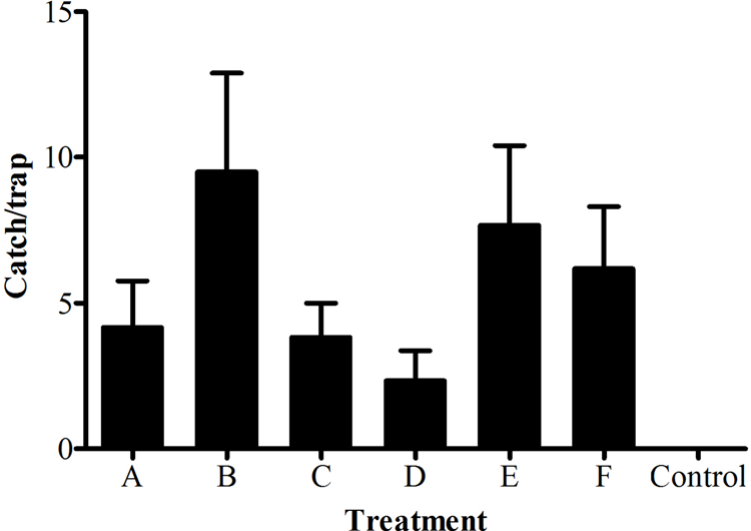
*Maruca vitrata* females caught in traps using different volatile components at field sites for one week. (A) Butanoic acid butyl ester, (B) Limonene, (C) 4-ethylpropiophenone, (D)1H-indol-4-ol, (E) Butanoic acid octyl ester, (F) 2-methyl-3-phenylpropanal. The significance of the difference between the blank lure control and volatiles groups was determined by Student's *t*-test. *P< 0.01.

In the fluorescence binding assays, six floral volatile components including butanoic acid butyl ester, limonene, 4-ethylpropiophenone, 1H-indol-4-ol, butanoic acid octyl ester and 2-methyl-3-phenylpropanal showed great binding affinities with MvitGOBP1-2 in 17 volatile odorant molecules. Therefore, the six compounds and blank lures were tested in the field trapping experiment. The trapping results demonstrated six floral volatile components of host plants could effectively attract some female moths and showed significant difference compared with the blank lure (Fig 7). Among them, limonene had the highest trapping effect in the field trapping experiments. These six compounds might be useful in baited traps for monitoring and forecasting of pests, which was similar to the sex pheromones of *M*. *vitrata*.

## Discussion

The legume pod borer is distributed throughout tropical and subtropical regions and management of *M*. *vitrata* relies mainly on chemical pesticides. However, abuse and residues of pesticides not only easily lead to increase of pest resistance, but also cause serious environmental pollution and decrease of their predator. Consequently, the high specificity and sensitivity of insect to floral volatiles and sex pheromones make them as effective biological control agents for population monitoring and mass trapping in integrated pest management (IPM) programs [31]. For instance, three sex pheromone components including (E, E)-10,12-Hexadecadienal, (E, E)-10, 12-Hexadecadienol and (E)-10-Hexadecenal have been applied to control this pest in the field [32–35]. The identification and functional analysis of odorant-binding proteins and pheromone-binding proteins in *M*. *vitrata* will provide new methods for us to control this pest through interfering their olfaction perception.

In this study, two GOBP genes, MvitGOBP1 and MvitGOBP2 were obtained from antennal tissue of *M*. *vitrata* and recombinant proteins were expressed in *E*. *coli*. The expression profile analysis revealed that *MvitGOBP1* and *MvitGOBP2* were expressed specifically at a very high level in the female and male antennae, which implies that *MvitGOBP1-2* is likely to be involved in chemoreception. The expression levels in the head (without antennae), thorax, abdomen, leg and wing of *M*. *vitrata* were significantly lower than antennae, which were consistent with other GOBPs of Lepidoptera species. Further, quantitative real-time PCR showed that the *MvitGOBP1-2* had different sex-biased expression patterns, with *MvitGOBP2* being highly male-biased (13.78-fold difference) and *MvitGOBP1* slightly female-biased (1.99-fold difference). In *Spodoptera exigua*, two GOBPs were approximately sex-equivalent (the absolute value < 1.90-fold difference) [36]. In *Orthaga achatina*, the transcription level of *OachGOBP2* in males was also higher than that in females, which was consistent with *MvitGOBP2* [37]. We suggest that the highly sex-biased *MvitGOBP1-2* possibly play different roles in host recognition of *M*. *vitrata*. Sequence alignment showed relatively high sequence identities with other insect GOBPs, especially CmedGOBPs (*C*. *medinalis*) and LstiGOBPs (*Loxostege sticticalis*). MvitGOBP1-2 have six highly conserved cysteine residues and conform to a common pattern within the OBPs: X_18_—Cys—X_30_—Cys—X_3_—Cys—X_42_—Cys—X_8-10_—Cys—X_8_—Cys—X_24-26_, which associated with other Lepidoptera species [23]. Interestingly, a seventh Cys residue follows the six Cys sequence and this characteristic has been found in *CmedGOBP1-2* from *C*. *medinalis* [38]. Phylogenetic analysis also showed that MvitGOBPs and CmedGOBPs are clustered in same group and showed their seventh Cys residues may have similar functions.

In addition, 17 electroantennogram-active compounds were identified from floral volatiles of *V*. *unguiculata* by gas chromatography-mass spectrometry and gas chromatography-electroantennography. These key volatile components including ester, ketone and aldehyde compounds showed different EAG responses and involved in host-plant detection of *M*. *vitrata*. In order to further clarify molecular mechanism of host recognition, the binding characteristics of MvitGOBP protein and host-plant volatiles were determined in present study. Among the 17 tested compounds, the binding activity of butanoic acid butyl ester, limonene, 4-ethylpropiophenone and 1H-indol-4-ol to MvitGOBP1 were best and displaced half 1-NPN from the MvitGOBP1/1-NPN complex at a ligand concentration of 20 mM. In our previous study, we got nine same volatile compounds from the odor profiles of two host plants *V*. *unguiculata* and *L*. *purpureus*, which showed obvious electrophysiological and olfactory behavioral responses [10]. Limonene and 1H-indol-4-ol as one of the same floral volatiles elicited high EAD responses and significantly attracted many *M*. *vitrata* females approaching the lure sources in the wind tunnel experiments. Binding abilities of limonene, butanoic acid butyl ester and 1H-indol-4-ol revealed that they should be involved in location of host-plants through binding MvitGOBP1 existed in olfactory sensilla from antenna of *M*. *vitrata*. MvitGOBP2 displayed the highest bind ability to 2-methyl-3-phenylpropanal which was the most abundant compound in both floral volatile blends and elicited high electrophysiological responses on antenna of *M*. *vitrata* (Fig 1). 2-methyl-3-phenylpropanal has been proved to attract significantly *M*. *vitrata* females in previous wind tunnel bioassays [10]. Butanoic acid octyl ester also had very great bind ability with MvitGOBP2, though its abundance was low and the EAG response was weaker than other plant volatiles. In addition, in the field trapping experiments, different numbers of female adults of *M*. *vitrata* were captured in traps that used six floral volatiles. The most female moths were trapped by Limonene, which it elicited weak EAD responses and evoked strong behavioral activity in the wind tunnel [10]. These results demonstrated that these six effective compounds may serve as attractants, while MvitGOBP1-2 might be used to recognize volatile odorants of host-plant flowers and involved in the selection of suitable oviposition sites in *M*. *vitrata*.

Our previous study suggested that female *M*. *vitrata* also could use other floral volatile components from *V*. *unguiculata* and *L*. *purpureus* to locate suitable hosts outside these six key odor compounds, though their binding capacities with MvitGOBPs was not great [10]. These floral volatile components might bind other OBPs from olfactory sensilla of *M*. *vitrata* antenna, which is essential for attraction and detection of host-plants. Generally speaking, the OBPs were large olfactory gene families and differentially expressed among diverse classes of sensilla respectively, which had unique odor specificities [25, 39]. Therefore, we infer that other OBPs might combine these volatile chemical molecules to exhibit different olfactory function in *M*. *vitrata*. Ligand binding capacity and key amino acid site of OBPs binding different floral volatile ligands from *V*. *unguiculata* need further study for illustrating olfactory molecular mechanism of *M*. *vitrata*. In conclusion, present and previous studies indicated that female *M*. *vitrata* might use these seventeen key floral volatiles from *V*. *unguiculata* to locate suitable hosts and artificial lure might be useful in exploring efficiency monitoring and integrated management strategies of the legume pod borer in the field.

## Supporting Information

S1 Fig (Fig 4A) Original data about expression levels of MvitGOBP1-2 in different tissues measured by real-time qPCR. (PZF)

S2 Fig (Fig 4B) Original data about expression levels of MvitGOBP2 in different tissues measured by real-time qPCR. (PZF)

S3 Fig (Fig 6A) Original data about binding curve and relative Scatchard plot in ligand-binding experiments. (PZF)

S4 Fig (Fig 6B–6E) Original data about competitive binding curves of different ligands to the MvitGOBP1. (PZF)

S5 Fig (Fig 6F–6I) Original data about competitive binding curves of different ligands to the MvitGOBP2. (PZF)

S6 Fig (Fig 7) Original data about field trap experiments of six key volatile components. (PZF)
